# Patient satisfaction after miraDry^®^ treatment for axillary hyperhidrosis. Results of an online patient survey after miraDry^®^ treatment to reduce excessive axillary sweating

**DOI:** 10.3205/iprs000188

**Published:** 2024-10-15

**Authors:** Ursula Tanzella, Klaus Ueberreiter, Armin Bell, Moritz A. Krapohl, Björn Dirk Krapohl

**Affiliations:** 1 Park-Klinik Birkenwerder, Birkenwerder, Germany; 2 Albrecht Ludwig University of Freiburg, Faculty of Medicine, Freiburg, Germany; 3 Department of Oral-maxillofacial, Reconstructive, and Plastic Surgery, Medical University Lausitz – Carl-Thiem, Cottbus, Germany

**Keywords:** hyperhidrosis, microwave therapy, miraDry®

## Abstract

Hyperhidrosis, with a prevalence of 1 to 2% of the population, primarily affects young people under 40 years of age. The individually perceived burden of odor and amount of sweat leads to a reduced quality of life.

In recent years, conservative and surgical measures have been used to treat hyperhidrosis. The miraDry^®^ method based on microwave technology is a non-invasive treatment that enables comparable results in terms of effectiveness while at the same time reducing the burden.

In the Park-Klinik Birkenwerder, 282 hyperhidrosis patients were treated with the miraDry^®^ method between 2017 and 2024. An online survey was conducted in May and June 2024. 220 patients were contacted, the results of 80 patients are available (response rate: 36.4%). Changes in the restrictions caused by increased sweating in various areas of life were asked before and after the treatment. In addition, the assessment of general quality of life before and after the treatment was compared.

There is a significant reduction in restrictions and a corresponding increase in quality of life after treatment with miraDry^®^.

Satisfaction with the method is high, which is reflected in a high recommendation rate of over 80%.

## Abstract (de)

Die Hyperhidrose betrifft mit einer Prävalenz von 1 bis 2 % der Bevölkerung vor allem junge Menschen unter 40 Jahren. Die individuell empfundene Belastung durch Geruch und Schweißmenge führt zu einer geminderten Lebensqualität.

Zur Behandlung der Hyperhidrose kamen in den vergangenen Jahren konservative und chirurgische Maßnahmen zur Anwendung. Die auf Mikrowellen-Technik basierende Methode miraDry^®^ stellt eine nicht-invasive Behandlung dar, welche hinsichtlich ihrer Wirksamkeit vergleichbare Ergebnisse bei gleichzeitig geringerer Belastung ermöglicht.

In der Park-Klinik Birkenwerder wurden zwischen 2017 und 2024 282 Hyperhidrose-Patienten mit der miraDry^®^-Methode behandelt. Im Mai und Juni 2024 wurde eine Online-Befragung durchgeführt. 220 Patienten wurden kontaktiert, es liegen die Antworten von 80 Patienten vor (Rücklaufquote: 36,4%). Erfragt wurden Veränderungen der Einschränkungen durch das vermehrte Schwitzen in verschiedenen Bereichen des Lebens vor und nach der Behandlung. Des Weiteren wurde die Einschätzung der allgemeinen Lebensqualität vor und nach der Behandlung verglichen.

Es zeigt sich eine deutliche Reduzierung der Einschränkungen und eine damit einhergehende Erhöhung der Lebensqualität nach der Behandlung mit miraDry^®^.

Die Zufriedenheit mit der Methode ist hoch, was sich in einer hohen Weiterempfehlungsrate von über 80% widerspiegelt.

## Introduction

Sweating is a physiological and useful process that originates from the eccrine sweat glands and is controlled by the sympathetic nervous system. It serves to regulate heat by releasing the watery fluid that is produced in the sweat glands onto the skin, creating a cooling effect through evaporation. However, if this useful function is decoupled from the respective situation and occurs, for example, spontaneously in an excessive form – i.e. as a malfunction – then this is referred to as hyperhidrosis. In affected patients, it is not an increase in the number or enlargement of the existing sweat glands, but an overstimulation of secretion. Why this overstimulation occurs in some people and not in others has not been conclusively clarified. It is currently assumed that the prevalence in the population is between 1 and 2%. It mainly affects younger people under the age of 40.

A typical patient‘s history of hyperhidrosis is as follows:

- Onset of symptoms during puberty (before age 25)
- Occurrence is arbitrary and uncontrollable
- Occurrence more than once a week
- No increased sweating at night
- Familial disposition is frequent

The predilection sites for primary idiopathic hyperhidrosis are the armpits, soles of the feet, palms of the hands, scalp/forehead and the inguinal area.

A classification of hyperhidrosis according to severity can be found in Table 1 (Tab. 1).

Hyperhidrosis poses a major challenge to the lives of those affected. They often feel severely restricted, which in turn can lead to professional and social stress and even social isolation.

The treatment of hyperhidrosis therefore has not only a health but also a social significance. The restriction of quality of life in a period of life that is normally characterized by a wealth of professional and social activities is a social problem that requires increased attention.

The treatment options include conservative, topical applications of, for example, aluminum hydroxide-containing deodorants, injections of botulinum toxin, interventions with heat in the form of radio frequency, microwave or focused ultrasound, and surgical interventions in the form of excision of the affected areas or subcutaneous curettage after previous liposuction.

The following presentation of the results of an online survey of patients from 2017 to 2024 at the Park-Klinik Birkenwerder (evaluation May to June 2024) relates exclusively to the treatment of axillary hyperhidrosis with the miraDry^®^ procedure.

## Materials and methods

### Technique of miraDry^®^ treatment

On the day of treatment, the affected area is marked in color using the iodine-starch test according to Minor [1]. Iodine solution in the form of Betaisadona^®^ is applied to the affected area and covered with starch powder. The steric re-formation of iodine and starch causes the affected area to darken on the skin. This discolored area is circled with a skin marker.

The area is then injected subcutaneously with local anesthetic so that the entire area is anaesthetized and “pumped up” (thickened and elevated).

The template with markings is then placed on the skin to match the size of the area to be treated and a non-permanent tattoo is applied to the skin. This tattoo serves as a pattern for the treatment points, which the microwave can then gradually act on. The treatment head is placed on the skin point by point under suction. The epidermis is cooled protectively while the microwave energy is delivered to the deeper skin layer in a controlled manner. The sweat glands are located in this layer and are thermally destroyed by the application of heat.

After the therapy, which lasts about an hour, the affected areas are cooled again and then padded with loose gauze compresses.

### Patient clientele and survey

At the Park-Klinik Birkenwerder, a total of 282 patients, 110 female (39%) and 172 male (61%) with hyperhidrosis were treated using the miraDry^®^ procedure between 2017 and 2024. The youngest patient was 19 years old, the oldest 50 years old. The average age was 37 years. Of 282 patients, 220 patients were asked to take part in the online survey and fill the questionnaire. We received 80 response forms, which corresponds to a response rate of 36.4%. The shortest period between treatment and survey was 3 months, the longest 5 years.

The online survey included questions about restrictions in:

1. private life (clothing, leisure activities, sports),
2. social and love life and
3. professional life.

The patients were able to give their individual assessment on a scale of 1 to 5 (1: slight restriction, 2: moderate restriction, 3: medium restriction, 4: severe restriction, and 5: very severe restriction).

The patients were also asked about their quality of life before and after treatment. The question about quality of life was also rated on a scale of 1 to 5 (1: slight, 2: moderate, 3: medium, 4: good, and 5: very good).

The respondents were able to provide information about whether they would recommend the method to others. A free text field also gave the respondents the opportunity to write in prose about their experiences before, during and after treatment.

## Results

In the following, the results of the online survey are presented.

In our patient group, 61.3% of patients have been suffering from the symptoms for 10 years or more, 28.7% for 5 years or more. 73.8% of patients had one miraDry^®^ treatment, 23.2% had two, and 3% had three. 87.5% of patients had already undergone conservative pretreatment using aluminum deodorants and/or botulinum toxin injections before miraDry^®^ therapy.

The following temporary complications occurred in our patient population:

- Temporary swelling
- Persistent swelling lasting more than 6 weeks
- Hematomas in the treated area
- Paraesthesia
- Local infection

**Figure 1:**
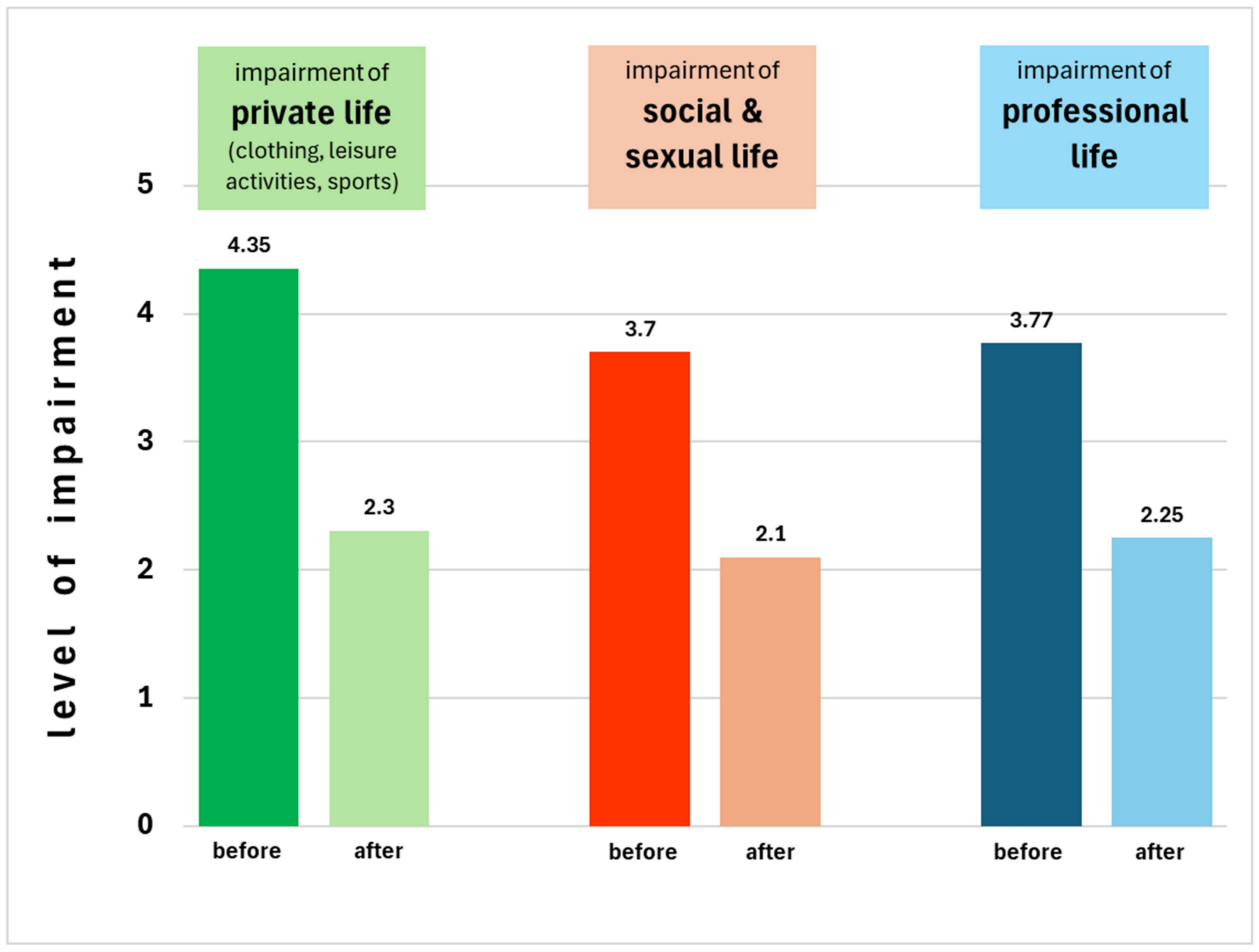
The graphic shows the level of impairment given by the patients for three different areas of life (green, red, blue) before (strong color) and after (1pale color) the miraDry^®^ treatment. We note a reduction. The reduction of impairment is seen in all three areas of life: private life, social and sexual life, as well as professional life.

Figure 1 (Fig. 1) depicts the comparison of the limitations before and after treatment in private life, social and sexual life, and professional life.

**Figure 2:**
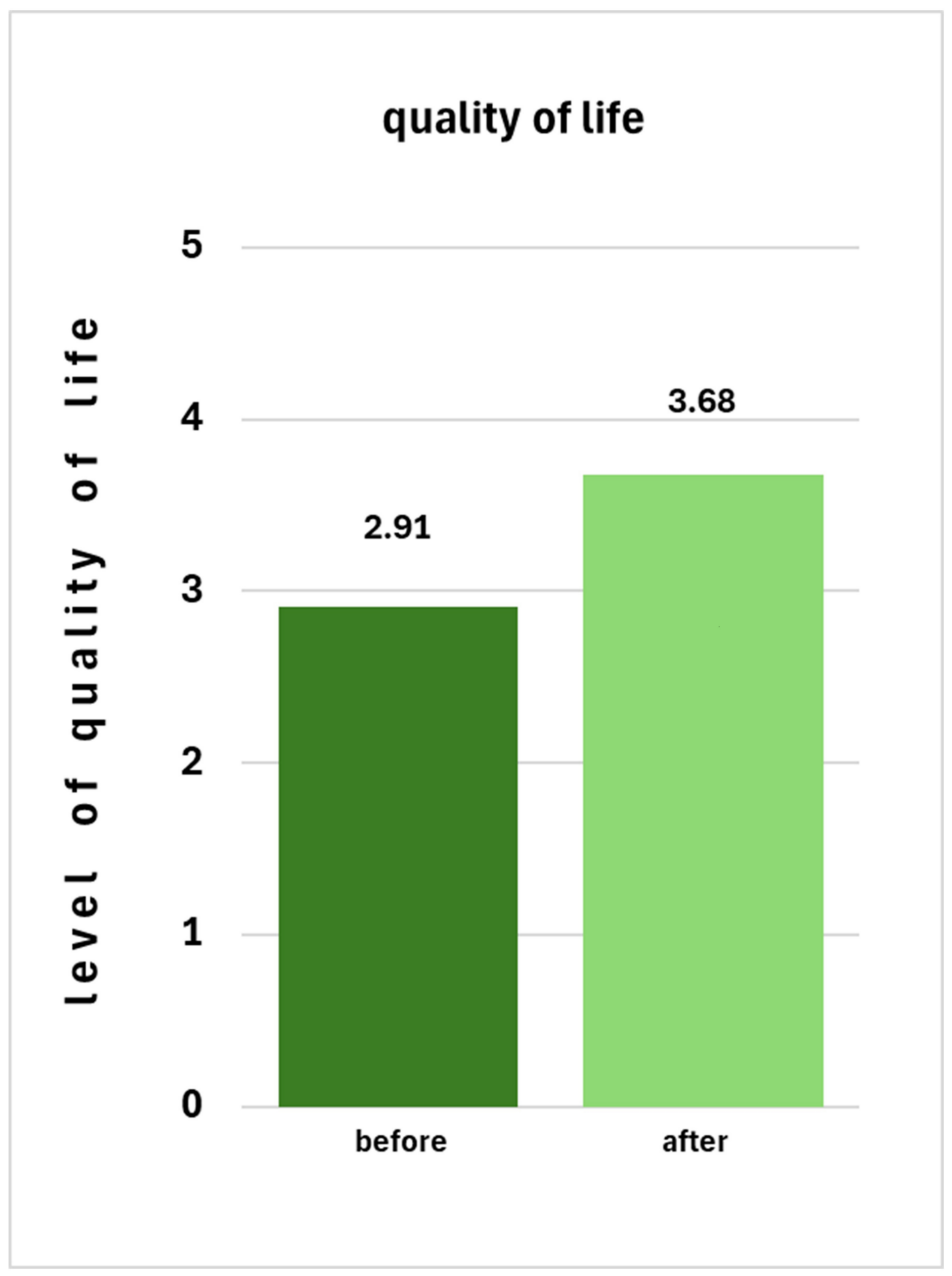
This diagram shows the change in the level of quality of life reported by the respondents using the score [1 to 5] before (dark green) and after (light green) miraDry^®^ treatment.

Figure 2 (Fig. 2) shows the comparison of the quality of life before and after treatment.

Figure 1 (Fig. 1) shows that the limitations in the areas of life surveyed are significantly reduced in intensity after miraDry^®^ treatment. This observation is also reflected in Figure 2 (Fig. 2), where we see an increase in quality of life after treatment with miraDry^®^ compared to before.

81.2% of the patients surveyed would recommend the treatment. 18.8% of the patients would not recommend the treatment or would only recommend it to a limited extent.

The comments in the free text field mainly reflect this proportion of the patients surveyed in their comments. These express disappointment about an insufficient effect or the need for a second treatment. Some patients state that the effect of the treatment had significantly diminished after a period of 2 years.

## Discussion

Hyperhidrosis, characterized by excessive sweating, represents a significant impairment of the quality of life for those affected [2], [3]. Traditional treatment methods such as drug treatments, topical applications of deodorants and surgical therapies such as liposuction in combination with curettage offer relief from symptoms, but have their own limitations [4], [5], [6], [7].

In the case of drug therapy, these are possible systemic side effects, in the case of topical applications, the limited effectiveness and in the case of liposuction with curettage, the possible postoperative complications such as cord formation and scarring [8].

For several years, the use of microwave technology in the form of miraDry^®^ has been established as a non-invasive treatment option [9], [10], [11], [12].

Compared to other methods that were used exclusively before the establishment of microwave therapy, miraDry^®^ is a non-invasive procedure that does not require surgery or medication and is therefore associated with fewer post-operative and systemic complications. The treatment is performed on an outpatient basis, which minimizes the overall burden on the patient [13], [14].

The effectiveness of miraDry^®^ has been confirmed in several clinical studies. In their study from 2012, Hong et al. reported an average reduction in sweat production of 82% after two treatments. The most common side effects were temporary swelling and redness, which, however, subsided within a few weeks [15].

Glaser et al. conducted a randomized controlled trial and found that 90% of patients reported a significant improvement in their symptoms after treatment [16].

To date, there are no long-term studies to fully assess long-term effectiveness and safety [17].

The evaluation of the patient survey in our patient population confirms the effectiveness already established in previous studies with a simultaneous low occurrence of complications. [18], [19] If these did occur, they were transient. Major complications such as permanent plexus injuries [20], [21] or serious infections [22] did not occur in our treated and follow-up patient population.

There is a clear improvement in living situations in all areas surveyed. Although the disabilities caused by sweating did not go back to zero in the surveyed areas, they did show a clear reduction. This led to an overall increase in the quality of life score after treatment.

Nevertheless, the method shows its limitations. This was particularly evident in the patient group who would not recommend the treatment or would only recommend it to a limited extent. In this group, disappointment about the less than 100% effect was predominant.

The respondents also negatively judged the fact that the treatment in some cases needed to be repeated until symptoms were sufficiently reduced.

## Conclusion

miraDry^®^ is an effective alternative to traditional hyperhidrosis therapies. It offers the advantage of a non-invasive treatment with a high success rate and a low incidence of complications. Compared to drug therapies and liposuction, miraDry^®^ shows a high level of patient acceptance and satisfaction.

In the explanatory discussion before treatment, it must be clearly communicated that a recurrence is possible, that several treatments may be required before the symptoms are relieved, and that the effect may diminish over the long term.

## Notes

### Competing interests

The authors declare that they have no competing interests.

## Figures and Tables

**Table 1:**
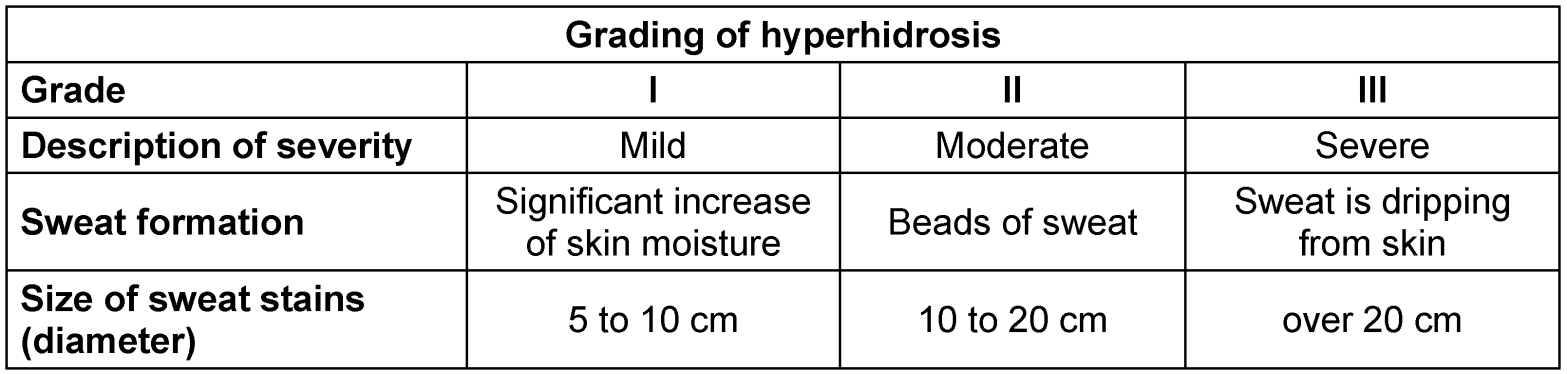
Grading of hyperhidrosis and clinical characteristics
